# Prone Position Effects in the Treatment of Covid-19 Patients

**DOI:** 10.22088/cjim.11.0.580

**Published:** 2020

**Authors:** Farzad Rahmani, Shiva Salmasi, Parisa Rezaeifar

**Affiliations:** 1 Emergency Medicine Research Team, Tabriz University of Medical Sciences, Tabriz, Iran; 2 Tuberculosis and Lung Disease Research Center, Tabriz University of Medical Sciences, Tabriz, Iran

**Dear Sir,**

COVID-19 is new type of virus that infects respiratory system (1). Every patient with acute severe respiratory disorder should be managed in the Intensive Care Unit (ICU) based on the recommendation of the Surviving Sepsis Campaign panel (2). About 19% of patients infected by COVID-19 suffer from hypoxic respiratory failure, and approximately 14% will develop severe infection, who require oxygen therapy, and 5% will require mechanical ventilation and ICU admission (3-4). A study revealed that among the 52 patients with a severe COVID-19 infection, 67% had acute respiratory distress syndrome (ARDS), 63.5%, 42%, and 56% took high-flow nasal cannula (HFNC), invasive mechanical ventilation, and noninvasive mechanical ventilation, respectively (5). 

Prone positioning is a conventional method to enhance oxygenation in Acute Respiratory Distress Syndrome (ARDS) patients who need mechanical ventilator (6). It is proven that oxygenation is significantly more beneficial in prone position compared to the supine position. Furthermore, numerous lines of evidence have confirmed that prone positioning could prevent lung injuries caused by ventilators (6-7). In supine position, the heart and its adjacent parts likely compress the central posterior parts of the lung. In prone position, the central anterior parts are compressed; as a result, increasing cardiac output and improving pulmonary respiration are among the advantages of prone positioning (8). According to the present theories, prone positioning, by reducing ventral alveolar expansion and dorsal alveolar collapse, results in ventilation that is more homogeneous. This results in reduction in the difference between dorsal and ventral transpulmonary pressures, reduction in lung compression, and enhancing perfusion (9). ARDS and severe hypoxemic patients (Pao2: Fio2 ratio<150 mm Hg, Fio2≥0.6, PEEP≥5 cmH_2_O) can benefit from prone positioning if early intervention is performed and the positioning would last in relatively long sessions (6). 

It is difficult to predict patients’ response to prone positioning since it has different forms among patients. However, numerous randomized trials and meta-analyses have shown that prone position in conjunction with a lung-protective strategy, when performed early and in sufficient duration, may improve survival rate among the patients with ARDS (10). Also, prone positioning reduced 28-d and 90-day mortality rates and extubation time and increased ventilator-free days (10). In the first week of infection, the patients with moderate to severe ARDS are recommended to receive ventilation in the prone position (10). Prone positioning reduced the mortality rate among the patients who were ventilated for at least 12 hours. In addition, prone positioning increased the risks of pressure sores and endotracheal tube obstruction (9).

**Prone Position Ventilation in COVID-19: **Oxygen therapy, high-flow nasal cannula, and non-invasive ventilation may reduce the necessity for endotracheal intubation and decrease ventilator-associated complications and mortality. Although non-invasive ventilation may help patients in a safe way, it can cause risks to the health care staff due to the presence of infected aerosol. Therefore, non-invasive ventilation may be employed as an early intervention for selected patients who are infected by COVID-19 with milder acute hypoxemic respiratory failure (4). The methods of prone positioning are not simplistic, and they require coordination between the healthcare team (6). The contraindications and complications of using prone positioning are presented in table 1. In addition, enteral nutrition via nasogastric or nasoduodenal tube can be continued during proning (9-10). 

 Proning of awake COVID-19 patients has come to be a progressively popular intervention. Additionally, there is a theory that improving oxygenation and, consequently decreasing the need for invasive ventilation may be attained by adopting proning in non-intubated, awake COVID-19 patients. A recent study on more than 600 COVID-19 patients found that the awake prone position had significant effect in improving oxygenation and pulmonary heterogeneity (11). Moreover, a retrospective observation study on 79 patients has stated that awake prone position combined with HFNC therapy could be applied safely and efficiently in severe COVID-19 patients, as well as it may lessen the conversion to critical illness and the requirement for tracheal intubation (12). Similarly, the results of the study by Despres et al. on six patients have shown that to avoid intubation in severe COVID-19 patients with spontaneous breathing, prone position combined with either high-flow nasal oxygen (HFNO) or conventional oxygen therapy could be recommended (13). In conclusion, by considering the complications and benefits of prone positioning ventilation, the use of this method for no less than 12 hours daily is a safe technique in lowering the mortality in patients with ARDS. Prior studies have shown that the survival rate after severe ARDS was significantly higher in the prone group. Hence, prone positioning can be suggested for the treatment of COVID-19 patients with induced severe ARDS (4,^6^).

**Table 1:** The contraindications and complications of using prone positioning

**Contraindications**		**Complications**
**Absolute**	**Relative**	
-Unmonitored or significantly increased intracranial pressure -Unstable vertebral fractures	-Difficult airway management -Tracheal surgery or sternotomy during the previous 15 d -New tracheostomy (less than 24 h) -Single anterior chest tube with air leaks -Serious facial trauma or facial surgery during the previous 15 d -Increased intraocular pressure -Hemodynamic instability or recent cardiopulmonary arrest -Cardiac pacemaker inserted in the last 2 d -Ventricular assist device -Intra-aortic balloon pump -Deep venous thrombosis treated for less than 2 d -Massive hemoptysis requiring an immediate surgical or interventional -Radiology procedure -Continuous dialysis -Severe chest wall lesions ± rib fractures -Recent cardiothoracic surgery/unstable mediastinum or open chest -Multiple trauma with unstabilized fractures -Femur, or pelvic fractures ± external pelvic fixation -Pregnant women -Recent abdominal surgery or stoma formation -Kyphoscoliosis -Advanced osteoarthritis or rheumatoid arthritis -Body weight greater than 135 kg	-Edema (facial, airway, limbs, thorax) -Pressure sores -Conjunctival hemorrhage -Compression of nerves and retinal vessels -Endotracheal tube dislocation (main stem intubation or non-scheduled extubation), obstruction or kinking -Airway suctioning difficulty -Transient hypotension or oxygen desaturation -Worsening gas exchange -Pneumothorax -Thoracic drain kinking or obstruction -Cardiac events -Inadvertent dislodging of Swan-Ganz catheter -Vascular catheter kinking or removal -Vascular catheter malfunction during continuous veno-venous -Hemofiltration -Deep venous thrombosis -Urinary bladder catheter or nasogastric feeding tube displacement -Enteral nutrition intolerance; vomiting; feeding complications -Need for increased sedation or muscle paralysis -Difficulty in instituting cardiopulmonary resuscitation
